# Improved generalized ComBat methods for harmonization of radiomic features

**DOI:** 10.1038/s41598-022-23328-0

**Published:** 2022-11-08

**Authors:** Hannah Horng, Apurva Singh, Bardia Yousefi, Eric A. Cohen, Babak Haghighi, Sharyn Katz, Peter B. Noël, Despina Kontos, Russell T. Shinohara

**Affiliations:** 1 Center for Biomedical Image Computing and Analysis (CBICA), Department of Radiology, University of Pennsylvania, Philadelphia, PA 19104 USA; 2 Penn Statistics in Imaging and Visualization Endeavor (PennSIVE), Department of Biostatistics, Epidemiology, and Informatics, University of Pennsylvania, Philadelphia, PA 19104 USA; 3 Laboratory for Advanced Computed Tomography Imaging, Department of Radiology, University of Pennsylvania, Philadelphia, PA 19104 USA

**Keywords:** Statistics, Prognostic markers

## Abstract

Radiomic approaches in precision medicine are promising, but variation associated with image acquisition factors can result in severe biases and low generalizability. Multicenter datasets used in these studies are often heterogeneous in multiple imaging parameters and/or have missing information, resulting in multimodal radiomic feature distributions. ComBat is a promising harmonization tool, but it only harmonizes by single/known variables and assumes standardized input data are normally distributed. We propose a procedure that sequentially harmonizes for multiple batch effects in an optimized order, called OPNested ComBat. Furthermore, we propose to address bimodality by employing a Gaussian Mixture Model (GMM) grouping considered as either a batch variable (OPNested + GMM) or as a protected clinical covariate (OPNested − GMM). Methods were evaluated on features extracted with CapTK and PyRadiomics from two public lung computed tomography (CT) datasets. We found that OPNested ComBat improved harmonization performance over standard ComBat. OPNested + GMM ComBat exhibited the best harmonization performance but the lowest predictive performance, while OPNested − GMM ComBat showed poorer harmonization performance, but the highest predictive performance. Our findings emphasize that improved harmonization performance is no guarantee of improved predictive performance, and that these methods show promise for superior standardization of datasets heterogeneous in multiple or unknown imaging parameters and greater generalizability.

## Introduction

Radiomics, defined as the high-throughput extraction of quantitative features from medical images, has emerged in recent years as both an alternative and complement to genomic data for applications in precision oncology by leveraging standard-of-care images to interrogate the whole tumor^1,2^. While such applications of radiomics are promising, they often require multicenter datasets to demonstrate sufficient statistical power and generalizability for clinical translation. However, imaging acquisition protocols often vary by institution in acquisition parameters, reconstruction, and post-processing. The resulting heterogeneous datasets are broadly equivalent clinically, but often contain unwanted variation due to technical factors that can interfere with downstream predictive analyses, resulting in reduced study reproducibility^3^. Examples of this problem include recent studies in computed tomography of the lung (CT) showing that reconstruction kernel and slice thickness as well as subsequent predictive analyses, as well as studies in magnetic resonance imaging (MRI) and positron emission tomography (PET) indicating acquisition parameters, site, and scanner can result in reduced feature reproducibility^4–9^.

Harmonization methods developed to solve this problem can be broadly separated into two groups based on their domain: the image domain or the feature domain^10^. Approaches in the image domain apply correction for differences associated with technical factors prior to feature extraction and include the standardizing protocols, developing more robust feature definitions, and image preprocessing^10,11^. However, these methods are often difficult to implement or require modification of existing guidelines for radiomic feature extraction. Generative deep learning approaches that translate images between “batches”, or images grouped by a particular parameter (i.e. different scanners) can also be classified as in the image domain. Examples of such methods include STAN-CT, where a generative adversarial network (GAN) is used to translate CT images by reconstruction kernel and slice thickness, and DeepHarmony, where a U-Net is used to translate MRI images by site^12,13^. While these approaches are promising, they require large datasets or specialized study designs and the transformations they apply are difficult to interpret.

Approaches in the feature domain apply correction after feature extraction, and include feature selection and batch effect correction methods^10^. Feature selection results in the elimination of features deemed to be non-robust to technical factors and can help alleviate collinearity, but it also can result in the loss of information that could prove useful in predictive analysis. Batch effect correction methods effectively standardize data following feature extraction without further loss of information, where batch effects are defined as non-biological factors that alter data.

One popular batch effect correction method is ComBat, a statistical harmonization method originally developed for genomics that can correct variation in imaging features due to imaging parameters by using empirical Bayes to estimate location and scale parameters^14,15^. Many recent radiomic studies have demonstrated that ComBat can harmonize radiomic features from different CT, MRI, and PET protocols and reduce the number of features with significantly different distributions attributable to batch effects^14–20^. While ComBat is fast, easy to use, and effective at small sample sizes, it has several limitations. The first is that ComBat assumes errors from standardized input data will follow a normal distribution, an assumption that does not hold when feature distributions are multimodal. While it has been claimed that ComBat can be applied to non-Gaussian distributions, there are not yet sufficient data to demonstrate its efficacy in the context of multimodal distributions^21^. The second is that ComBat requires all batch effects and clinical covariates be known for effective correction or preservation of variation. This problem is not unique to radiomics–recent work in genomics has also sought to address the problem of batch effect correction in the setting of unknown subtypes^22^. Lastly, the standard implementation of ComBat is only able to harmonize by a single batch effect at a time when datasets are often heterogeneous in more than one parameter.

In our previous work, we introduced two methods of addressing these limitations–Nested ComBat to harmonize by more than one imaging parameter and Gaussian Mixture Model (GMM) ComBat to estimate the scan groupings associated with an unknown covariate to remove bimodality^23^. However, Nested ComBat was unable to outperform standard ComBat, likely due to bimodal feature distributions and suboptimal determination of harmonization order^23^. While GMM ComBat did successfully address the problem of bimodality, it did not address other batch variables affecting the data^23^. Crucially, because the scan groupings attributable to an unknown covariate are estimated purely from the shape of the distribution in GMM ComBat, whether the covariate is a clinical variable (whose effect must be preserved) or an imaging parameter (whose effect must be removed) is unknown.

In this work, we further develop Nested ComBat into OPNested ComBat which selects an optimal batch effect harmonization order and results in improved feature performance compared with standard ComBat (Table 1). In addition, we explore novel methods of accounting for unknown covariates causing multimodal feature distributions. To address this, we fully incorporate the GMM grouping from GMM ComBat into OPNested ComBat in two distinct approaches: first as an unknown imaging parameter to be corrected (OPNested + GMM ComBat), and second as an unknown clinical variable to be protected (OPNested − GMM ComBat). These updated iterative ComBat methods promise better standardization of radiomics data that are affected by multiple batch effects and/or exhibit bimodal feature distributions (Table 1). We then demonstrate the utility of these approaches on radiomics features extracted from publicly available lung CT images for removing variation associated with CT device manufacturer, spatial resolution to reconstruction kernel, and the use of intravenous contrast agents.

**Table 1:** Summary of methods introduced in this work.

	Description	Use Case
Optimized Permutation Nested ComBat (OPNested ComBat)	Updated version of Nested ComBat that optimizes harmonization order by selecting the permutation associated with the smallest number of features with statistically significant differences in distribution due to batch effects	Datasets heterogeneous in multiple imaging parameters
OPNested + GMM ComBat	Generalizes OPNested by adding a mixture model grouping to the list of imaging parameters for OPNested ComBat harmonization	Datasets heterogeneous in multiple imaging parameters with bimodality assumed to be associated with an imaging parameter
OPNested − GMM ComBat	Generalizes OPNested by adding a mixture model grouping to the list of clinical covariates protected during OPNested ComBat harmonization	Datasets heterogeneous in multiple imaging parameters with bimodality assumed to be associated with a clinical covariate of interest

## Results

### Harmonization performance evaluation

**Figure 1:**
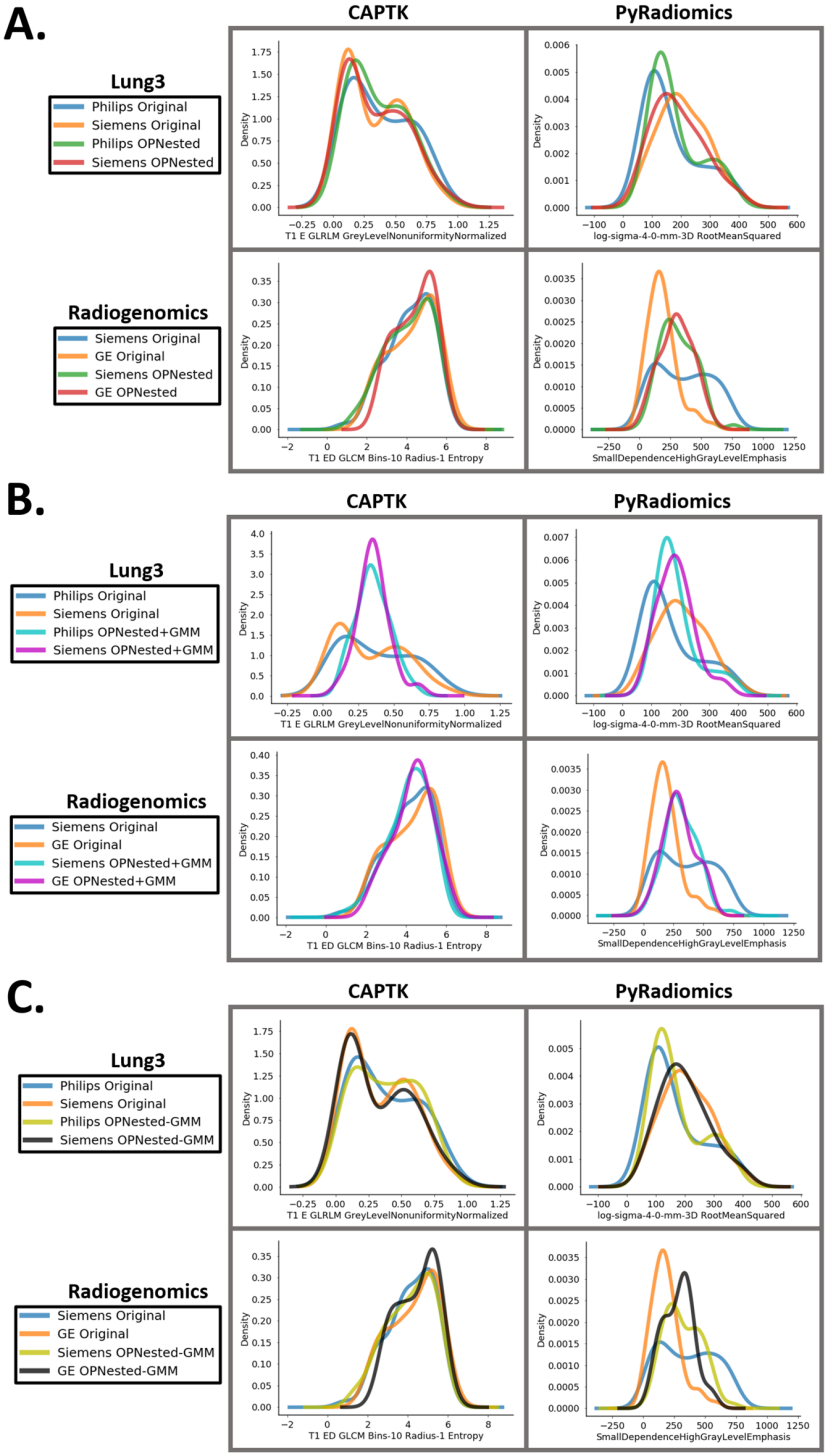
(**A)** Representative kernel density plots for the original features and after applying OPNested ComBat. (**B)** Representative kernel density plots for the original features and after applying OPNested + GMM ComBat (**C)** Representative kernel density plots for the original features and after harmonizing with OPNested − GMM ComBat. Kernel density plots represent ComBat results separated by the batch variable manufacturer, and plots for representative features whose distributions best visually demonstrate the effects of GMM ComBat were selected by screening all the feature distributions before and after harmonization. Harmonization should result in more similar feature distributions.

The results of standard ComBat, OPNested ComBat, OPNested + GMM ComBat, and OPNested − GMM ComBat are shown in Table 2 and Fig. 1. We use the difference in percentage of features with significant differences in distribution relative to the original data as a measure of effect size when evaluating and comparing harmonization performance because correlation between features prevents the use of a binomial test for significant differences in proportions. Percentages differences were computed by subtracting the percentage of features with statistically significant differences in distribution attributable to batch effects for one feature set (e.g., features harmonized by OPNested ComBat) from another feature set (e.g., original features). Negative differences in percentage are indicative of improved harmonization performance. OPNested ComBat generally tended to outperform standard ComBat in harmonization performance across all datasets (percentage difference for OPNested ComBat subtracted from standard ComBat: −10.8%, −49.8%, −24.5%. −7.6% for Lung3/CAPTK, Lung3/PyRadiomics, Radiogenomics/CAPTK, and Radiogenomics/PyRadiomics, respectively, when split by contrast enhancement). This was an improvement over the previous version where OPNested ComBat was only able to demonstrate comparable performance when compared to standard ComBat^23^. The application of OPNested + GMM ComBat resulted in the greatest reduction in the percentage of features with significant differences associated with known batch effects across all datasets (percentage difference for OPNested + GMM ComBat subtracted from Original: −21.5%, −41.7%, −2.6%, −51.7% for Lung3/CAPTK, Lung3/PyRadiomics, Radiogenomics/CAPTK, and Radiogenomics/PyRadiomics, respectively, when split by contrast enhancement). OPNested + GMM ComBat also successfully reduced the percentage of features with significant differences in distribution due to the inferred GMM grouping (percentage difference for OPNested + GMM subtracted from Original: −50%, −59.6%, −42.2%, 65.1% for Lung3/CAPTK, Lung3/PyRadiomics, Radiogenomics/CAPTK, and Radiogenomics/PyRadiomics, respectively, when split by contrast enhancement), a reduction that was much smaller in magnitude in the features harmonized with OPNested-GMM ComBat (percentage difference for OPNested–GMM ComBat subtracted from Original: −6.9%, −8.4%, −3.9%, −6.5% for Lung3/CAPTK, Lung3/PyRadiomics, Radiogenomics/CAPTK, and Radiogenomics/PyRadiomics, respectively, when split by contrast enhancement). Harmonization with OPNested − GMM ComBat also resulted in a smaller reduction in the percentage of features with significant differences due to the known individual batch effects when compared to OPNested + GMM ComBat and OPNested ComBat in all datasets (percentage difference for OPNested − GMM ComBat subtracted from Original: −9.8%, −37.2%, −15.7%, −9.6% for Lung3/CAPTK, Lung3/PyRadiomics, Radiogenomics/CAPTK, and Radiogenomics/PyRadiomics, respectively, when split by contrast enhancement).

**Table 2:** Percentage of features out of the original number of features with significantly (*p* < 0.05) different distributions attributable to batch effects in the original features and after applying standard ComBat, OPNested ComBat, OPNested + GMM ComBat, and OPNested – ComBat. Order indicates order of batch effects used in sequential harmonization for multiple batch effects. GMM groupings are generated by selecting the best model out of a set of GMMs estimated from each feature, thus GMM feature indicates the feature corresponding to the best model used to generate the final GMM grouping for all features.

	CE	Spatial Resolution	Manufacturer	GMM Class
**Lung3/CAPTK**^**a**^				
Original	22.5%	27.5%	56.9%	92.2%
ComBat	15.7%	16.7%	47.1%	
**OPNested**	4.9%	10.8%	31.4%	
**OPNested + GMM**	1%	6.9%	4.9%	42.2%
**OPNested − GMM**	12.7%	17.6%	50%	85.3%
**Lung3/PyRadiomics**^**b**^				
Original	41.9%	51.2%	65.1%	84.7%
ComBat	51.2%	24.7%	27.7%	
**OPNested**	1.4%	28.8%	26.5%	
**OPNested + GMM**	0.2%	21.6%	22.6%	25.1%
**OPNested − GMM**	4.7%	20%	42.8%	76.3%
**Radiogenomics/CAPTK**^**c**^				
Original	31.4%	52.9%	20.6%	85.3%
ComBat	38.2%	40.2%	50%	
**OPNested**	13.7%	21.6%	56.9%	
**OPNested + GMM**	8.8%	16.7%	11.8%	43.1%
**OPNested − GMM**	15.7%	31.4%	53.9%	81.4%
**Radiogenomics/PyRadiomics**^**d**^				
Original	57.7%	74%	44.9%	78.6%
ComBat	22.3%	31.6%	36%	
**OPNested**	14.7%	29.8%	22.6%	
**OPNested + GMM**	6%	39.5%	16.5%	13.5%
**OPNested − GMM**	48.1%	67%	18.4%	72.1%

^a^Nested Order: Manufacturer, Spatial Resolution, CE, Nested + GMM Order: Spatial Resolution, GMM, Manufacturer, CE, Nested – GMM Order: CE, Manufacturer, Spatial Resolution, GMM Feature: T1_E_GLRLM_ShortRunLowGreyLevelEmphasis.

^b^Nested Order: CE, Spatial Resolution, Manufacturer, Nested + GMM Order: Spatial Resolution, CE, GMM, Manufacturer, Nested-GMM Order: Manufacturer, CE, Spatial Resolution, GMM Feature: ldmn.

^c^Nested Order: Manufacturer, CE, Spatial Resolution, Nested + GMM Order: GMM, CE, Spatial Resolution, Manufacturer, Nested-GMM Order: Manufacturer, CE, Spatial Resolution, GMM Feature: T1_ED_GLRLM_Bins-10_Radius-1_ShortRunLowGreyLevelEmphasis.

^d^Nested Order: Spatial Resolution, Manufacturer, CE, Nested + GMM Order: Spatial Resolution, GMM, Manufacturer, CE, Nested – GMM Order: CE, Spatial Resolution, Manufacturer, GMM Feature: JointEnergy.

The results of statistical testing on the features residualized on the clinical variables with a linear regression are shown in Table S1. While the percentages of features with significant differences in distribution due to imaging parameters are smaller than the values in the non-residualized features, the trends regarding the best-performing harmonization approach remain the same. In addition, statistical testing for significant differences in distribution due to imaging parameters in the principal component scores used as predictors in method evaluation are shown in Table S2. Principal components generated from the OPNested ComBat approach contained no such significant differences in two out of the four datasets, while the OPNested + GMM ComBat yielded the same result in one of the datasets. OPNested, OPNested + GMM, and OPNested − GMM ComBat better reduced the number of principal components with significant differences in distributions due to imaging parameters when compared to standard ComBat.

### Outcome predictive performance evaluation

**Figure 2:**
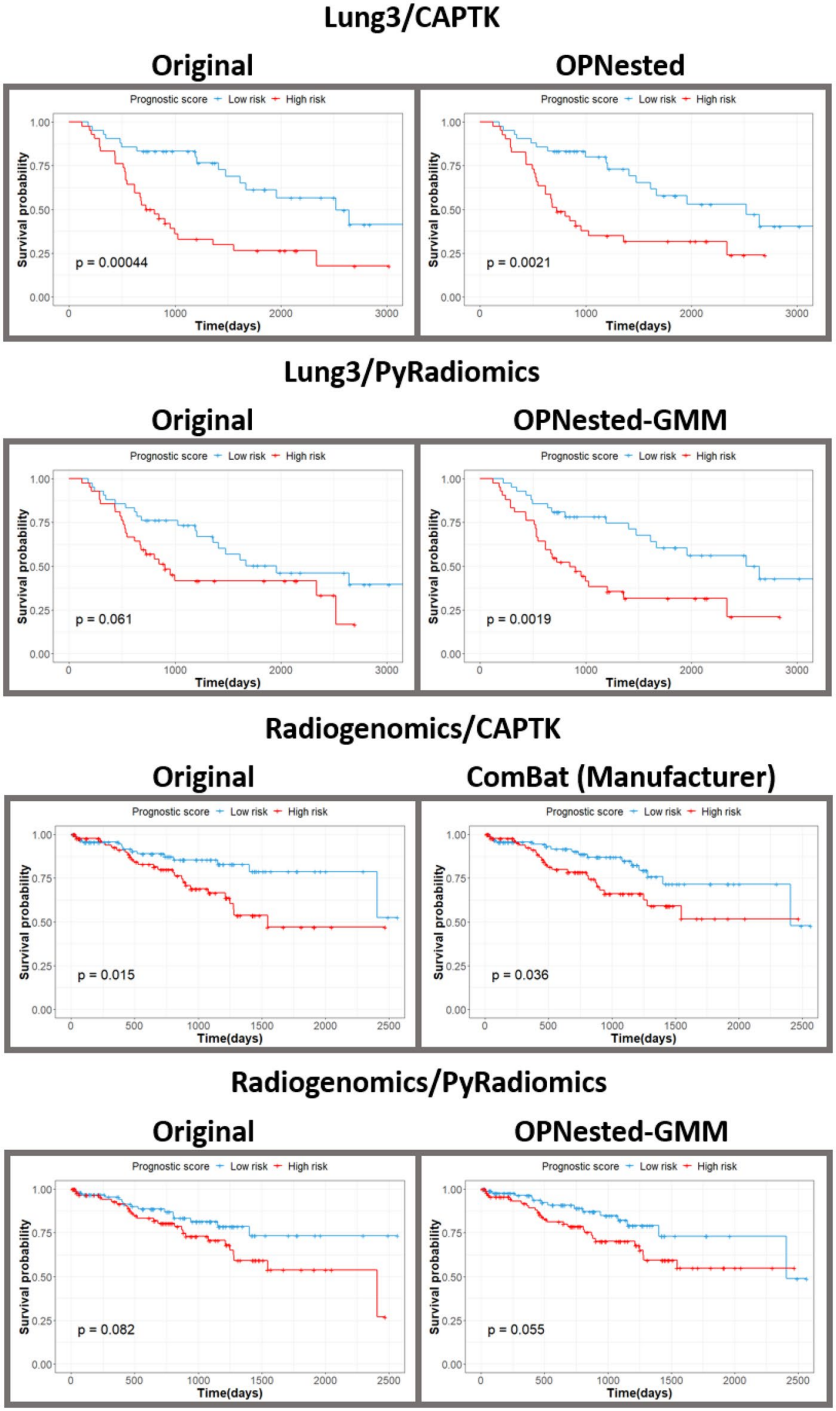
In-sample Kaplan–Meier curves fitted on the original features and the harmonization approach with the highest c-statistic.

The results of survival analyses completed with the original and harmonized features are shown in Table 3 and Fig. 2. Both the original and harmonized features from all datasets, apart from Radiogenomics/PyRadiomics, resulted in significant (*p* < 0.05) log rank test *p* values for Kaplan–Meier curve separation. In the Radiogenomics/PyRadiomics data, Kaplan–Meier curves from both the original and harmonized data did not result in a significant *p* value from the log rank test (Fig. 2).

**Table 3:** C-statistics and 95% confidence intervals (CI) (over 2000 iterations) for fivefold cross-validated Cox proportional hazard models built exclusively from imaging-based features decomposed with PCA (with no added clinical covariates) to predict survival, and log-rank *p* values for Kaplan–Meier curve separation. ComBat (Manufacturer) indicates data was harmonized by manufacturer with ComBat.

	fivefold CV c-statistic	95% CI	Log-rank *p* value
**Lung3/CAPTK**			
Original	0.59	[0.53, 0.64]	0.0004
ComBat (Manufacturer)	0.62	[0.56, 0.67]	0.014
**OPNested**	0.62	[0.57, 0.67]	0.0021
**OPNested + GMM**	0.50	[0.43, 0.56]	0.027
**OPNested-GMM**	0.59	[0.53, 0.64]	0.0011
**Lung3/PyRadiomics**			
Original	0.62	[0.57, 0.67]	0.061
ComBat (Manufacturer)	0.64	[0.59, 0.68]	0.0004
**OPNested**	0.65	[0.60, 0.60]	0.0032
**OPNested + GMM**	0.61	[0.55, 0.66]	0.0036
**OPNested-GMM**	0.65	[0.60, 0.69]	0.0019
**Radiogenomics/CAPTK**			
Original	0.58	[0.52, 0.62]	0.015
ComBat (Manufacturer)	0.58	[0.51, 0.64]	0.036
**OPNested**	0.54	[0.46, 0.61]	0.019
**OPNested + GMM**	0.56	[0.49, 0.61]	0.012
**OPNested-GMM**	0.55	[0.47, 0.61]	0.029
**Radiogenomics/PyRadiomics**			
Original	0.63	[0.59, 0.67]	0.082
ComBat (Manufacturer)	0.59	[0.54, 0.63]	0.13
**OPNested**	0.59	[0.54, 0.63]	0.12
**OPNested + GMM**	0.58	[0.52, 0.63]	0.41
**OPNested-GMM**	0.59	[0.54, 0.64]	0.055

OPNested ComBat and standard ComBat (on a single batch effect, i.e., manufacturer) exhibited the highest c-statistic (0.62/*p* = 0.22, *p* = 0.24, respectively) in the Lung3/CAPTK dataset, while OPNested + GMM had the lowest c-statistic (0.50/*p* = 0.01) (Table 3). OPNested + GMM also had the lowest c-statistic in the Lung3/PyRadiomics (0.61/*p* = 0.41), while OPNested and OPNested–GMM ComBat had the highest c-statistics (0.65/*p* = 0.17, * p* = 0.21, respectively). In the Radiogenomics/CAPTK data, ComBat harmonization by manufacturer was associated with the highest c-statistic (0.58/*p* = 0.47) to match the c-statistic from the original features, while OPNested had the lowest such statistic (0.54/*p* = 0.29). In the Radiogenomics/PyRadiomics data, OPNested + GMM ComBat had the lowest c-statistic (0.58/*p* = 0.06), but no harmonization approach was able to match or exceed the c-statistic from the original features (0.63).

In addition, survival analyses were performed for both (a) the original features and (b) features harmonized using each of the different approaches where features with a statistically significant difference in distribution observed with at least one imaging parameter were removed from the dataset (DROP) (Table S3, Figure S1). This differs from the analyses outlined above, in which all features were retained regardless of statistical testing indicating significant differences in distribution attributable to imaging parameters. In the Lung3 dataset, the additional dropping of features did not improve c-statistics over the standard ComBat and OPNested − GMM approaches in the CapTK and PyRadiomics features, respectively. In the Radiogenomics dataset, dropping features did not improve the c-statistics over the OPNested − GMM approach in the CapTK data, but did improve the c-statistic in the original features (0.65) to outperform OPNested − GMM ComBat without dropping features in the PyRadiomics data.

### Associations between batch variables and clinical covariates

Results from the chi-squared testing for independence and point biserial correlation testing to detect associations between clinical variables and imaging parameters (including the GMM groupings) are shown in Table 4. In the Lung3 data, survival was significantly associated with manufacturer (*p* = 0.016). In the Radiogenomics data, manufacturer was significantly associated with sex (*p* < 0.001), smoking (*p* = 0.001), and histology classification (*p* = 0.011). Spatial resolution was also associated with smoking (*p* = 0.048). In addition, the GMM grouping generated from the CapTK features were found to be significantly associated with gender (*p* = 0.043) and histology classification (*p* = 0.013).

**Table 4:** P values from the chi-squared test for independence (between categorical variables) and point biserial correlation test (between categorical and continuous variables) to detect association between clinical variables and imaging parameters, as well as GMM groupings. Red fill indicates *p* values below the 0.05 threshold for significance.

	Manufacturer	Spatial resolution	CE	CAPTK GMM	PyRadiomics GMM
Lung3					
Survival	0.016	0.080	0.611	0.961	0.998
Histology	0.098	0.912	0.059	0.809	0.522
Stage	0.627	0.821	0.448	0.188	0.110
Gender	0.623	0.739	0.988	0.139	0.295
Survival time (months)	0.051	0.101	0.273	0.754	0.622
Radiogenomics					
Survival	0.147	0.571	0.295	0.455	0.885
Gender	< 0.001	0.081	0.125	0.043	0.842
Smoking	0.001	0.048	0.082	0.058	0.553
Histology	0.011	0.116	0.601	0.013	0.154
Survival time (days)	0.485	0.647	0.689	0.832	0.729

In addition, results from the chi-squared test for independence to detect associations between imaging parameters within each dataset are shown in Table S4. In the Lung3 data, manufacturer was found to be associated with spatial resolution (*p* < 0.001) as well as the GMM groupings generated from the CapTK (*p* = 0.016) and PyRadiomics data (*p* = 0.01). In the Radiogenomics data, manufacturer was associated with the GMM grouping generated from the CapTK data (*p* = 0.044). Spatial resolution was also found to be associated with manufacturer (*p* < 0.001), contrast enhancement (*p* < 0.001), and the GMM groupings generated from the PyRadiomics data (*p* < 0.001). In both datasets, the GMM grouping generated from the CapTK data was associated with the grouping generated from the PyRadiomics data (*p* < 0.001 for Lung3 and Radiogenomics). Note that the GMM groupings for each dataset were generated from different features, as GMM groupings are generated by selecting the best model out of a set of GMMs estimated from each feature.

## Discussion

While standard ComBat can effectively harmonize by a single imaging parameter under strong normality assumptions, the heterogeneity of imaging datasets in more than one imaging parameter necessitated the development of Nested ComBat to sequentially harmonize by each batch effect^23^. However, the previous version of Nested ComBat failed to surpass standard ComBat in harmonization performance in our two lung CT datasets (Lung3 and Radiogenomics), likely due to a combination of bimodal feature distributions and poorly optimized harmonization order^23^. In this work, we update Nested ComBat into OPNested ComBat to optimize the order of batch effects in sequential harmonization by testing all possible orders and selecting the best one, resulting in superior performance when compared to the previous version. However, it can be observed that bimodal feature distributions remain bimodal following harmonization with OPNested ComBat, violating a key assumption made by ComBat that the residuals from the standardized input data will be normally distributed and limiting harmonization performance (Fig. 1).

In many instances, bimodality may be due to an unknown variable not measured in the study. This variable could be a nuisance imaging parameter or a variable of interest. In our previous study, we introduced the use of a GMM to estimate the scan groupings for a hidden variable from the distribution of an imaging variable^23^. In this work, we use two methods of fully integrating the GMM grouping into OPNested ComBat. In OPNested + GMM ComBat, we assume that the hidden variable is an imaging parameter with associated variation that must be corrected. Harmonization with OPNested + GMM ComBat resulted in the lowest percentages of features with significant differences in distribution due to the known imaging parameters as well as the hidden parameter implied by the GMM grouping (Table 2). This is likely because the inclusion of the GMM grouping in the list of batch effects for sequential harmonization by OPNested ComBat eliminates bimodality in the feature distributions, as can be visually observed in Fig. 1. However, while OPNested + GMM ComBat demonstrated the best harmonization performance, using radiomic features harmonized with OPNested + GMM ComBat resulted in reduced predictive performance when compared to features harmonized OPNested ComBat (Table 3).

These findings indicate that the GMM grouping assumed to be a technical variable unassociated with biological variation could in fact be a biological variable. Thus, correcting variation associated with the GMM grouping resulted in the removal of biological variation of interest and reduced predictive performance. This explanation is further supported by the observation that the GMM grouping generated from the Radiogenomics CapTK features was found to be significantly associated with the biological variables of gender and histology, and that the percentages of features with significant differences in distribution due to technical variables decreased when the features were residualized on biological variables (Table 4, Table S1). Another possible explanation is that because imaging parameters were generally associated with outcome as a consequence of study design, the removal of variation associated with those imaging parameters reduce predictive performance. This hypothesis is supported by the finding that manufacturer was significantly associated with survival outcome in the Lung3 data and gender, smoking, and histology in the Radiogenomics data (Table 4). Future work will include using multivariate modeling to estimate the scan groupings associated with unknown covariates as a function of both imaging parameters and clinical variables to better determine the true cause of the observed bimodal feature distributions. Future work could also involve curation of datasets with more detailed clinical and image acquisition information to better determine whether the GMM grouping is biological or technical in nature.

The findings from OPNested + GMM ComBat resulted in the development of OPNested − GMM ComBat, in which we assume that the hidden variable causing the bimodality and driving the GMM grouping is a biological variable with associated variation that must instead be protected during harmonization. OPNested − GMM ComBat resulted in worse harmonization performance when compared to OPNested + GMM and OPNested ComBat, but consistently demonstrated higher predictive performance than OPNested + GMM ComBat and had the highest c-statistic in two out of the four datasets (Table 3). In addition, while there were more principal components with significant differences in distribution due to imaging parameters computed from features harmonized with the OPNested − GMM approach when compared with the OPNested + GMM approach, the features harmonized with the OPNested − GMM approach still demonstrated superior predictive performance (Table S2). These findings further support the hypothesis that the generated GMM grouping is in some way associated with outcome, potentially biologically or as a technical variable imbalanced across outcome. As an example, consider a hypothetical study in which most patients without progression-free survival were imaged using a Siemens scanner. Because of this imbalance, the technical variable of manufacturer becomes associated with the outcome of progression-free survival.

The hypothesized association between technical factors and clinical covariates is supported by chi-squared and point biserial correlation testing for association between the known clinical variables and imaging parameters (including the GMM groupings), which indicate that the GMM grouping generated from the Radiogenomics/CapTK data were significantly associated with two clinical variables (Table 4). In addition, the reduced harmonization performance observed in OPNested − GMM ComBat when compared to OPNested + GMM ComBat could be because the unknown covariates estimated by the GMM groupings are also associated with technical variables. Statistical testing indicated that GMM groupings in both datasets and toolkits were significantly associated with known technical variables (Table 4). This association could result in unwanted variation associated with technical variables being preserved when the GMM grouping is listed as a protected covariate, possibly resulting in reduced harmonization performance.

Another possible hypothesis for the reduced predictive performance from OPNested + GMM ComBat is that the process of iterative harmonization applies too many transformations to the data, resulting in distortion of the original signal. Future harmonization algorithms for multiple imaging parameters could address this problem by completing harmonization through a single transform as opposed to an iterative procedure.

While removing features with significant differences in distribution associated with imaging parameters is a potential method for removing any batch effects that remain post-harmonization, our findings demonstrate that this failed to improve predictive performance in two out of the four datasets (Table S3). This is likely because the process of dropping features can result in a loss of relevant information, particularly when imaging parameters are associated with clinical variables of interest. Future work could include developing superior batch effect detection metrics that incorporate biological associations to serve as a better criterion for feature selection.

In addition, while ComBat can remove batch effects impacting the mean and variance of quantitative features extracted from medical images, it does not effectively address batch effects in the covariance of these features^24^. This hypothesis is supported by our finding that principal components from harmonized data still contained significant differences attributable to technical factors (Table S2). Chen et al. have developed an improved version of ComBat, CovBat, that is better able to address covariance batch effects^24^. Future work could include application of CovBat to our radiomic feature datasets and development of iterative and mixture model extenstions of CovBat to improve harmonization performance.

In this work, we have improved the Nested ComBat algorithm into OPNested ComBat to optimize the order of sequential harmonization and improve harmonization performance. We also introduce two new iterative ComBat methods, OPNested + GMM ComBat and OPNested − GMM ComBat, that fully integrate the GMM grouping introduced in our previous work into OPNested ComBat. While assuming the GMM grouping was attributable to an imaging parameter in OPNested + GMM ComBat resulted in the best harmonization performance, assuming the GMM grouping was caused by a clinical variable in OPNested − GMM ComBat resulted in the best predictive performance. Both methods show promise for improving performance in secondary analyses and improving study reproducibility. However, the disconnect between harmonization and predictive performance serves as a reminder to the radiomics community that while harmonization can often improve the performance of predictive models by removing unwanted variation due to batch effects, it can also result in reduced predictive performance when a “batch effect” is in fact a “clinical variable” or closely associated with one. In addition, studies with additional, larger, datasets are needed to further validate our findings.

## Material and methods

### Statistical testing

The Anderson–Darling (AD) test was used to assess for general differences in distribution associated with imaging parameters. The AD test was selected over the Wilcoxon-Rank Sum and Kruskal–Wallis tests given that many of the feature distributions appeared multimodal. It was selected over the Kolmogorov–Smirnov test because it enables testing of more than two batches associated with a particular imaging parameter and its increased weighting on the tails of a distribution. The percentage of radiomic features out of the original number of features with detected significant (*p* < 0.05) differences in distribution associated with an individual imaging parameter was used to measure the ability of ComBat and its iterative variations to remove variation caused by the corresponding imaging parameter.

In addition, the chi-squared test for independence was used to assess for associations between batch effects and categorical clinical variables. Similarly, the point biserial correlation test was used to test for associations between batch effects and continuous clinical variables.

### OPNested, OPNested + GMM, and OPNested − GMM ComBat

**Figure 3:**
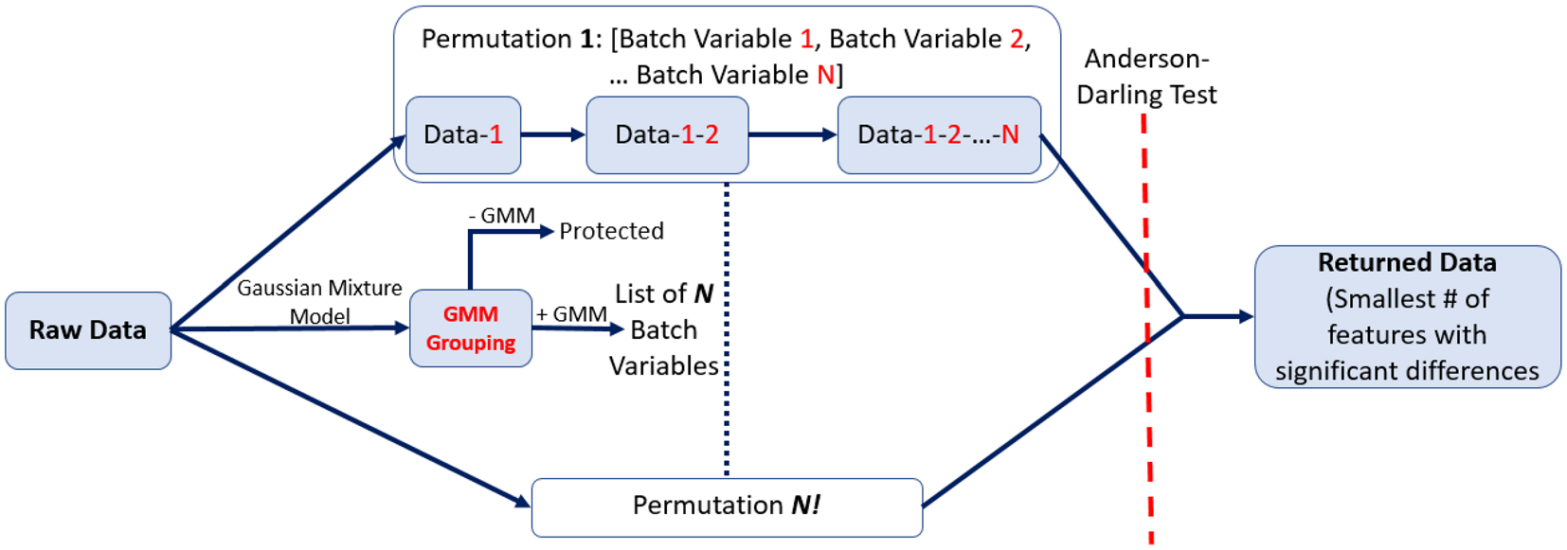
Workflow for the OPNested, OPNested + GMM, and OPNested − GMM ComBat implementations for sequential harmonization given two batch effects. Red denotes a batch effect, while the dash indicates that the data has been harmonized by a particular batch effect (i.e., Data-1 means the data has been harmonized by batch effect 1, Data-1-2 means Data-1 has been harmonized again by batch effect 2, etc.).

In this study, we refine the methods from our previous work to optimize the harmonization order for iterative ComBat methods enabling sequential harmonization by multiple batch effects^23^. We initialize with a list of batch effects and the original radiomic features as the input data, just as in the previous version of Nested ComBat^23^. However, in the updated version of OPNested ComBat we generate a list of all possible harmonization orders by computing a list of all permutations of the initialized batch effect list (Fig. 3). At each iteration, the original input data were sequentially harmonized with ComBat with order given by the permutation corresponding to the iteration (i.e. harmonization by the first batch effect in the permutation, then harmonizing the resulting harmonized data by the second batch effect in the permutation, etc.). The resulting harmonized feature sets, each corresponding to a different order/permutation, were each assessed for significant differences in distribution attributable to each batch effect using the AD test. The harmonized feature set with the lowest total number of features with significant differences in distribution across all batch effects was selected as the final output.

In our previous work, we introduced GMM ComBat in which a two-component Gaussian mixture model (GMM) is used to identify scan groupings likely split by an unknown covariate assumed to be an imaging parameter from the observed feature distribution^23^. However, we did not add the ability to additionally harmonize by multiple imaging parameters, limiting its performance. In this study, we develop two methods of fully integrating GMM ComBat into OPNested ComBat. In OPNested + GMM ComBat, we maintain the assumption that the scan groupings estimated by the GMM are caused by an imaging parameter and add them to the list of batch effects for sequential harmonization in OPNested ComBat. The data are thus harmonized by both the pre-specified known imaging parameters as well as the GMM grouping. In OPNested − GMM ComBat, we instead assume that the scan groupings estimated by the GMM are caused by a clinical covariate. The GMM grouping is consequently added to the list of variables to be protected during OPNested ComBat harmonization to ensure the corresponding variation is not affected.

Source code for implementing the updated version of OPNested ComBat algorithm, as well as the new implementations of OPNested + GMM ComBat and OPNested − GMM ComBat, can be found at https://github.com/hannah-horng/opnested-combat.

### Datasets

We used two datasets publicly available from NCI’s The Cancer Imaging Archive (TCIA) to evaluate the performance of our harmonization methods, Lung3 and Radiogenomics^25–27^. Studies for the collection of both datasets were approved by the Institutional Review Boards at their respective institutions, and both datasets were fully de-identified prior to being made publicly available^26,27^. All datasets and methods used in this work were compliant with relevant guidelines and regulations. Further information regarding case selection can be found in our preceding manuscript, while imaging parameter information is shown in Table 5 and Table S5 and patient demographics are shown in Table S6^23,25–27^. The 3D tumour volume on these images was segmented by a board-certified, fellowship-trained thoracic radiologist with 16 years of clinical experience (S.K.) using the semi-automated ITK-SNAP software (v 3.6.0)^28^. Features from lung tumor volumes segmented from both imaging datasets were extracted with the Cancer Imaging Phenomics Toolkit (CapTK) (102 features) and the PyRadiomics software library (430 features), resulting in a total of four sets of features^29,30^. A table of the extracted features can be found in Table S7–S8.

**Table 5:** Case counts by batch effect for the Lung3 and Radiogenomics datasets.

	Lung3	Radiogenomics
Non contrast-enhanced	34	102
Contrast-enhanced	50	91
Low spatial resolution	49	91
High spatial resolution	35	102
Siemens	37	54
General Electric	–	139
Philips	47	–

### ComBat

All ComBat analyses used the *neuroComBat* Python package, which harmonizes data by a single batch effect^16^. The performance of the standard implementation of ComBat was assessed by applying separate harmonization by each of the three batch effects (contrast enhancement, spatial resolution, manufacturer) (Table 5). In the Lung3 dataset, the clinical variables of death event, histology, stage, gender, and survival were protected. In the Radiogenomics dataset, the clinical variables of death event, histology, gender, smoking status, and days were preserved.

### Method evaluation

Principal component analysis was used to generate ten radiomic principal components (PCs) from the original CapTK and PyRadiomics features in the Lung3 and Radiogenomics datasets and features harmonized with all harmonization methods. The total number of predictors was capped at 5 out of 10 in the CapTK features and 4 out of 10 in the PyRadiomics features to capture 85% of the variance, which follows the statistical rule of thumb of approximately one predictor per event (45 deaths in Lung3 and 40 deaths in Radiogenomics) to prevent model overfitting.

Each set of principal components was used in a five-fold cross-validated multivariate Cox proportional hazards model (2000 iterations) to compute the concordance index (c-statistic), which measures the ability of the models to predict survival. Confidence intervals were constructed by taking the 2.5% and 97.5% quantiles from the 2000 iterations of the cross-validated Cox models. We also built a model on the complete dataset to evaluate Kaplan–Meier performance in separating participants above versus below the median prognostic score. The log-rank test was used to statistically compare Kaplan–Meier curves. All models included imaging features only and did not include additional clinical variables. A bootstrap approach was used to obtain *p* values indicating statistically significant increases and decreases in predictive performance (quantified using the c-statistic) of post-harmonized features compared to the original features.

## Supplementary Information

Supplementary Information.

## Data Availability

The datasets analyzed in this work (Lung3 and Radiogenomics) are publicly available from NCI’s The Cancer Imaging Archive (TCIA) [https://wiki.cancerimagingarchive.net/display/Public/NSCLC-Radiomics-Genomics, https://wiki.cancerimagingarchive.net/display/Public/NSCLC+Radiogenomics]^25–27^.
