# Supplementary material for: Improved generalized ComBat methods for harmonization of radiomic features
Source: Sci Rep. 2022 Nov 8;12:19009. doi: 10.1038/s41598-022-23328-0 (PMC9643436; doi:10.1038/s41598-022-23328-0)
Supplement: Supplementary file 1 — Supplementary Information. [file 41598_2022_23328_MOESM1_ESM.docx]

**Improved generalized ComBat methods for harmonization of radiomic features**

**Hannah Horng^1,2^, Apurva Singh^1^, Bardia Yousefi^1^, Eric A. Cohen^1^, Babak Haghighi^1^, Sharyn Katz^1^, Peter B. Noël^3^, Despina Kontos^1*^, Russell T. Shinohara^2*^**

^1^Center for Biomedical Image Computing and Analysis (CBICA), Department of Radiology, University of Pennsylvania, Philadelphia, PA, 19104

^2^ Penn Statistics in Imaging and Visualization Endeavor (PennSIVE), Department of Biostatistics, Epidemiology, and Informatics, University of Pennsylvania, Philadelphia, PA, 19104

^3^ Laboratory for Advanced Computed Tomography Imaging, Department of Radiology, University of Pennsylvania, Philadelphia, PA, 19104

^*^ Co-senior authors and corresponding authors: [Russell.Shinohara@pennmedicine.upenn.edu](mailto:Russell.Shinohara@pennmedicine.upenn.edu), [Despina.Kontos@pennmedicine.upenn.edu](mailto:Despina.Kontos@pennmedicine.upenn.edu)

**SUPPLEMENTARY INFORMATION**

**Lung3/CAPTK**

|  | CE | Spatial Resolution | Manufacturer | GMM  (T1_E_GLRLM_  ShortRunLow  GreyLevel  Emphasis) |
| --- | --- | --- | --- | --- |
| Original | 6.9% | 6.9% | 26.5% | 84.3% |
| ComBat | 8.8% | 7.8% | 2% |  |
| Nested | 2% | 0% | 1% |  |
| Nested  + GMM | 1% | 0% | 2% | 22.5% |
| Nested  – GMM | 1% | 0% | 12.7% | 59.8% |

**Lung3/PyRadiomics**

|  | CE | Spatial Resolution | Manufacturer | GMM (Idmn) |
| --- | --- | --- | --- | --- |
| Original | 33.3% | 34.7% | 38.8% | 64.7% |
| ComBat | 37.4% | 23% | 6.3% |  |
| Nested | 0.2% | 4.2% | 0.5% |  |
| Nested + GMM | 0% | 2.8% | 0.5% | 7% |
| Nested – GMM | 1.2% | 3.7% | 2.8% | 57.9% |

**Radiogenomics/CAPTK**

|  | CE | Spatial Resolution | Manufacturer | GMM  (T1_ED_GLRLM_  Bins-10_Radius-1_ShortRun  LowGrey  LevelEmphasis |
| --- | --- | --- | --- | --- |
| Original | 15.7% | 52.9% | 26.5% | 83.3% |
| ComBat | 16.7% | 52.9% | 27.5% |  |
| Nested | 12.7% | 11.8% | 33.3% |  |
| Nested + GMM | 9.8% | 9.8% | 1% | 29.4% |
| Nested – GMM | 12.7% | 22.5% | 22.5% | 83.3% |

**Radiogenomics/PyRadiomics**

|  | CE | Spatial Resolution | Manufacturer | GMM (Joint  Energy) |
| --- | --- | --- | --- | --- |
| Original | 46.5% | 64.7% | 40.7% | 72.1% |
| ComBat | 48.1% | 63.7% | 18.1% |  |
| Nested | 8.8% | 21.9% | 14.7% |  |
| Nested + GMM | 2.1% | 36.5% | 14% | 9.1% |
| Nested - GMM | 21.6% | 60.2% | 12.3% | 70.9% |

**Table S1**. Percentage of features out of the original number of features with significantly (p < 0.05) different distributions attributable to batch effects in the original features and after applying standard ComBat, Nested ComBat, Nested+GMM ComBat, and Nested-GMM ComBat residualized using a linear regression model with clinical covariates as predictors.

**Lung3/CAPTK**

|  | CE | | Spatial Resolution | Manufacturer |
| --- | --- | --- | --- | --- |
| Original | | C5 | C3 | C1 |
| ComBat | | C3, C5 | C3 | None |
| Nested | | None | C3 | None |
| Nested + GMM | | None | C3 | C5 |
| Nested – GMM | | None | C3 | C1 |

**Lung3/PyRadiomics**

|  | CE | Spatial Resolution | Manufacturer |
| --- | --- | --- | --- |
| Original | C1, C3, C4 | C1, C2, C3 | C1, C3 |
| ComBat | C1, C3, C4 | C2, C3 | C3 |
| Nested | None | None | None |
| Nested + GMM | None | None | C4 |
| Nested – GMM | None | None | C2, C4 |

**Radiogenomics/CAPTK**

|  | CE | Spatial Resolution | Manufacturer |
| --- | --- | --- | --- |
| Original | C3, C4 | C1, C2, C3, C4 | C4, C5 |
| ComBat | C3, C4 | C3, C4 | C4 |
| Nested | None | C4 | C2 |
| Nested + GMM | None | None | None |
| Nested – GMM | None | C4 | C2 |

**Radiogenomics/PyRadiomics**

|  | CE | Spatial Resolution | Manufacturer |
| --- | --- | --- | --- |
| Original | C1, C2, C3 | C1, C2, C3 | C1, C2, C3, C4 |
| ComBat | C1, C2, C3 | C1, C2, C3, C4 | C4 |
| Nested | None | None | None |
| Nested + GMM | C4 | C4 | C4 |
| Nested – GMM | C4 | C1, C2 | C1 |

**Table S2.** Principal components with significant differences (p < 0.05) in distribution attributable to batch effects for the original features and after applying standard Combat, Nested ComBat, Nested+GMM ComBat, and Nested-GMM ComBat. An entry C*N* indicates that the *N*th component has significant differences in distribution due to batch effects. The total number of components was 5 for CapTK features and 4 for PyRadiomics features.

**Lung3/CAPTK**

|  | 5-fold CV c-statistic | | 95% CI | Log-rank p-value |
| --- | --- | --- | --- | --- |
| Original + DROP | | 0.60 | [0.55, 0.65] | 0.0038 |
| ComBat + DROP (Manufacturer) | | 0.63 | [0.56, 0.68] | 0.0002 |
| Nested + DROP | | 0.60 | [0.54, 0.65] | 0.074 |
| Nested+GMM + DROP | | 0.54 | [0.46, 0.62] | 0.0041 |
| Nested-GMM + DROP | | 0.59 | [0.52, 0.65] | 0.0037 |

**Lung3/PyRadiomics**

|  | 5-fold CV c-statistic | 95% CI | Log-rank p-value |
| --- | --- | --- | --- |
| Original + DROP | 0.61 | [0.56, 0.65] | 0.053 |
| ComBat + DROP (Manufacturer) | 0.64 | [0.59, 0.69] | 0.0003 |
| Nested + DROP | 0.63 | [0.58, 0.68] | 0.0018 |
| Nested+GMM + DROP | 0.59 | [0.53, 0.63] | 0.0076 |
| Nested-GMM + DROP | 0.65 | [0.59, 0.69] | 0.0025 |

**Radiogenomics/CAPTK**

|  | 5-fold CV c-statistic | 95% CI | Log-rank p-value |
| --- | --- | --- | --- |
| Original + DROP | 0.58 | [0.52, 0.63] | 0.0069 |
| ComBat + DROP (Manufacturer) | 0.58 | [0.52, 0.64] | 0.063 |
| Nested + DROP | 0.58 | [0.51, 0.63] | 0.11 |
| Nested+GMM + DROP | 0.55 | [0.49, 0.61] | 0.36 |
| Nested-GMM + DROP | 0.58 | [0.53, 0.62] | 0.0048 |

**Radiogenomics/PyRadiomics**

|  | 5-fold CV c-statistic | 95% CI | Log-rank p-value |
| --- | --- | --- | --- |
| Original + DROP | 0.64 | [0.60, 0.68] | 0.095 |
| ComBat + DROP (Manufacturer) | 0.60 | [0.54, 0.63] | 0.062 |
| Nested + DROP | 0.55 | [0.48, 0.61] | 0.043 |
| Nested+GMM + DROP | 0.57 | [0.50, 0.62] | 0.029 |
| Nested-GMM + DROP | 0.54 | [0.47, 0.59] | 0.17 |

**Table S3.** C-statistics and 95% confidence intervals (CI) (over 200 iterations) for 5-fold cross-validated Cox proportional hazard models built from harmonized data, and log-rank p-values for Kaplan-Meier curve separation. ComBat (Manufacturer) indicates data was harmonized by manufacturer with ComBat. DROP indicates that all features with a statistically significant difference in distribution observed with at least one imaging parameter were removed from the dataset.

**
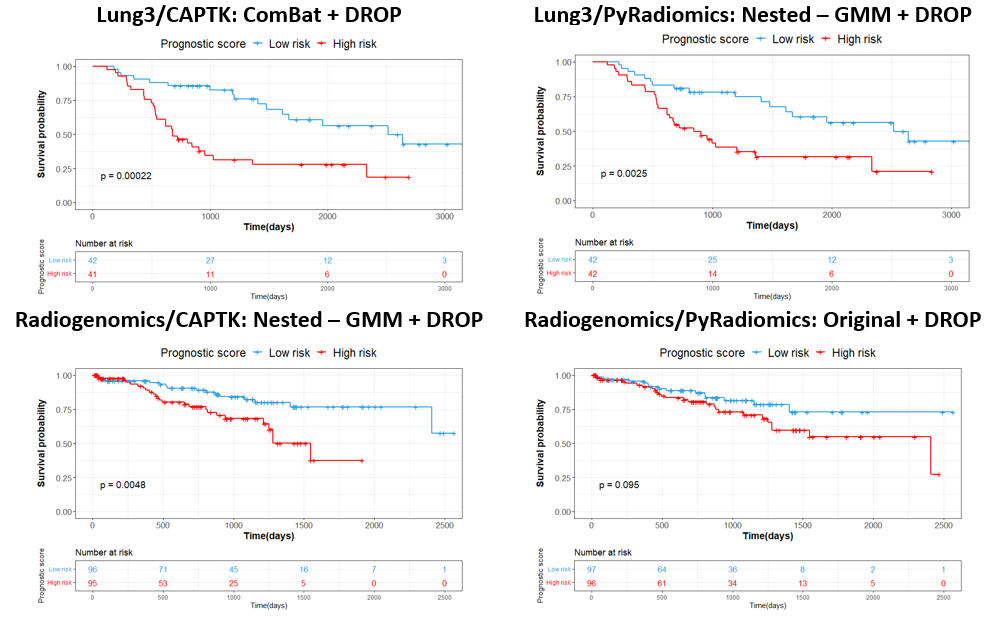
**

**Figure S1**. In-sample Kaplan-Meier curves fitted on the original features and the harmonization approach with the highest c-statistic following application of the DROP procedure. DROP indicates that all features with a statistically significant difference in distribution observed with at least one imaging parameter were removed from the dataset.

**Lung3**

| **Parameter 1** | **Parameter 2** | **P-Value** |
| --- | --- | --- |
| Manufacturer | Spatial Resolution | 1.10E-06 |
| Manufacturer | CE | 0.147 |
| Manufacturer | CAPTK GMM | 0.016 |
| Manufacturer | PyRadiomics GMM | 0.010 |
| Spatial Resolution | CE | 0.121 |
| Spatial Resolution | CAPTK GMM | 0.599 |
| Spatial Resolution | PyRadiomics GMM | 0.553 |
| CE | CAPTK GMM | 0.357 |
| CE | PyRadiomics GMM | 0.340 |
| CAPTK GMM | PyRadiomics GMM | 3.46E-09 |

**Radiogenomics**

| **Parameter 1** | **Parameter 2** | **P-Value** |
| --- | --- | --- |
| Manufacturer | Spatial Resolution | 4.17E-12 |
| Manufacturer | CE | 0.459 |
| Manufacturer | CAPTK GMM | 0.044 |
| Manufacturer | PyRadiomics GMM | 0.145 |
| Spatial Resolution | CE | 1.50E-05 |
| Spatial Resolution | CAPTK GMM | 0.435 |
| Spatial Resolution | PyRadiomics GMM | 1.37E-10 |
| CE | CAPTK GMM | 0.106 |
| CE | PyRadiomics GMM | 0.057 |
| CAPTK GMM | PyRadiomics GMM | 7.10E-05 |

**Table S4**. P-values from the chi-squared test for independence to detect association between imaging parameters within the Lung3 and Radiogenomics datasets. Red fill indicates p-values below the 0.05 threshold for significance.

|  | Lung3 | Radiogenomics |
| --- | --- | --- |
| Peak Kilovoltage | 120-140 kVp | 80-140 kVp |
| Tube Current | 33-463 mA | 100-750 mA |
| Slice Thickness | 1.5-5 mm | 0.625-3 mm |
| Reconstruction Diameter | 308-700 mm | 306-500 mm |
| Reconstruction Kernels | Phillips  A, B, C, D  Siemens  B30f, B30s, B40f, B60f, B70s | General Electric  BONE, BONEPLUS, LUNG, SOFT, STANDARD  Siemens  B19f, B20f, B25f, B31f, B31s, B40f, B45f, B45s, B50f, B60f, B70f |

**Table S5**. CT acquisition parameters for the Lung3 and Radiogenomics datasets.

**Lung3**

| Feature | Subject Counts |
| --- | --- |
| Gender  Male  Female | 57  27 |
| Survival  Yes  No | 39  45 |
| Histology  Adenocarcinoma  Squamous Cell Carcinoma  Other | 40  32  12 |
| Combined Stage  I  II  II  Unknown | 38  25  12  9 |

**Radiogenomics**

| Feature | Subject Counts |
| --- | --- |
| Gender  Male  Female | 128  65 |
| Survival  Yes  No | 152  41 |
| Histology  Adenocarcinoma  Squamous Cell Carcinoma  Other | 159  30  4 |
| Smoking  Nonsmoker  Former  Current | 119  42  32 |

**Table S6**. Patient demographics for the Lung3 and Radiomics datasets.

| Feature Category | Feature Name |
| --- | --- |
| Intensity | *Coefficient of Variation*  *Energy*  *Inter Quartile Range*  *Kurtosis*  *Maximum*  *Mean*  *Mean Absolute Deviation*  *Median*  *Median Absolute Deviation*  *Minimum*  *Mode*  *Ninetieth Percentile*  *Quartile Coefficient of Variation*  *Range*  *Root Mean Square*  *Skewness*  *Standard Deviation*  *Sum Tenth Percentile*  *Variance* |
| Histogram | *Frequency*  *Coefficient of Variation*  *Energy*  *Entropy*  *Fifth Percentile*  *Fifth Percentile Mean*  *Inter Quartile Range*  *Kurtosis*  *Mean*  *Mean Absolute Deviation*  *Median*  *Median Absolute Deviation*  *Mode*  *Ninetieth Percentile*  *Ninety Fifth Percentile*  *Ninety Fifth Percentile Mean*  *Quartile Coefficient of Variation*  *Robust Mean Absolute Deviation*  *Root Mean Square*  *Seventy Fifth Percentile*  *Skewness*  *Standard Deviation*  *Sum*  *Tenth Percentile*  *Twenty Fifth Percentile*  *Uniformity*  *Variance* |
| Volumetric | *Pixels*  *Volume* |
| Morphologic | *Eccentricity*  *Ellipse Diameter*  *Elongation*  *Equivalent Spherical*  *Perimeter*  *Equivalent Spherical Radius*  *Flatness*  *Largest Component Size*  *Number of Pixels*  *Perimeter*  *Physical Size*  *Roundness* |
| Grey level run length matrix (GLRLM) | *Grey Level Non- Uniformity Normalized*  *Grey Level Non-Uniformity*  *Grey Level Variance*  *High Grey Level Run Emphasis*  *Long Run Emphasis*  *Long Run High Grey Level Emphasis*  *Long Run Low Grey Level Emphasis*  *Low Grey Level Run Emphasis*  *Run Entropy*  *Run Length Non- Uniformity Normalized*  *Run Length Non- Uniformity*  *Run Length Variance*  *Run Percentage*  *Short Run Emphasis*  *Short Run High Grey Level Emphasis*  *Short Run Low Grey Level Emphasis*  *Total Runs* |
| Grey level size zone matrix (GLSZM) | *Grey Level Mean*  *Grey Level Non- Uniformity*  *Grey Level Non-Uniformity Normalized*  *Grey Level Variance*  *High Grey Level Emphasis*  *Large Zone Emphasis*  *Large Zone High Grey Level Emphasis*  *Large Zone Low Grey Level Emphasis*  *Low Grey Level Emphasis*  *Small Zone Emphasis*  *Small Zone High Grey Level Emphasis*  *Small Zone Low Grey Level Emphasis*  *Zone Percentage*  *Zone Size Entropy*  *Zone Size Mean*  *Zone Size Non- Uniformity*  *Zone Size Non-Uniformity Normalized*  *Zone Size Variance* |
| Neighboring grey tone difference matrix (NGTDM) | Busyness  *Coarseness*  *Complexity*  *Contrast*  *Strength* |

**Table S7**. Table of CAPTK features used in analysis.

| Feature Category | Feature Name |
| --- | --- |
| Intensity  *All intensity features were also calculated for LoG-sigma 5mm, 4.5mm, 4mm, 3.5mm, 3mm, 2.5mm, 2mm, 1.5mm, 1mm, 0.5mm and for Wavelet LLH, LHL, LHH, HLL, HLH, HHL, HHH, LLL | *Energy*  *Total Energy*  *Entropy*  *Minimum*  *10^th^ percentile*  *90^th^ percentile*  *Maximum*  *Mean*  *Median*  *Interquartile Range*  *Range*  *Mean Absolute Deviation (MAD)*  *Robust Mean Absolute Deviation (rMAD)*  *Root Mean Squared (RMS)*  *Skewness*  *Kurtosis*  *Variance*  *Uniformity* |
| Shape | *Elongation*  *Flatness*  *Least Axis Length*  *Major Axis Length*  *Maximum 2D Diameter-Column*  *Maximum 2D Diameter-Row*  *Maximum 2D Diameter-Slice*  *Maximum 3D Diameter*  *Minor Axis Length*  *Sphericity*  *Surface Area*  *Surface Volume Ratio*  *Volume* |
| Grey level co-occurrence matrix (GLCM) | *Autocorrelation*  *Joint Average*  *Cluster Prominence*  *Cluster Shade*  *Cluster Tendency*  *Contrast*  *Correlation*  *Difference Average*  *Difference Entropy*  *Difference Variance*  *Joint Energy*  *Joint Entropy*  *Informational Measure of Correlation (IMC) 1*  *Informational Measure of Correlation (IMC) 2*  *Inverse Difference Moment (IDM)*  *Maximal Correlation Coefficient (MCC)*  *Inverse Difference Moment Normalized (IDMN)*  *Inverse Difference (ID)*  *Inverse Variance*  *Maximum Probability*  *Sum Entropy*  *Sum of Squares* |
| Grey level size zone matrix (GLSZM) | Small Area Emphasis (SAE)  Large Area Emphasis (LAE)  Grey Level Non-Uniformity (GLN)  Grey Level Non-Uniformity Normalized (GLNN)  Size-Zone Non-Uniformity (SZN)  Size-Zone Non-Uniformity Normalized (SZNN)  Zone Percentage (ZP)  Grey Level Variance (GLV)  Zone Variance (ZV)  Zone Entropy (ZE)  Low Grey Level Zone Emphasis (LGLZE)  High Grey Level Zone Emphasis (HGLZE)  Small Area Low Grey Level Emphasis (SALGLE)  Small Area High Grey Level Emphasis (SAHGLE)  Large Area Low Grey Level Emphasis (LALGLE)  Large Area High Grey Level Emphasis (LAHGLE) |
| Grey level run length matrix (GLRLM) | Short Run Emphasis (SRE)  Long Run Emphasis (LRE)  Grey Level Non-Uniformity (GLN)  Grey Level Non-Uniformity Normalized (GLNN)  Run Length Non-Uniformity (RLN)  Run Length Non-Uniformity Normalized (RLNN)  Run Percentage (RP)  Grey Level Variance (GLV)  Run Variance (RV)  Run Entropy (RE)  Low Grey Level Run Emphasis (LGLRE)  High Grey Level Run Emphasis (HGLRE)  Short Run Low Grey Level Emphasis (SRLGLE)  Short Run High Grey Level Emphasis (SRHGLE)  Long Run Low Grey Level Emphasis (LRLGLE)  Long Run High Grey Level Emphasis (LRHGLE) |
| Neighboring grey tone difference matrix (NGTDM) | Coarseness  Contrast  Contrast 2  Complexity  Strength |
| Grey level dependence matrix (GLDM) | Large Dependence Emphasis (LDE)  Grey Level Non-Uniformity (GLN)  Dependence Non-Uniformity (DN)  Dependence Non-Uniformity Normalized (DNN)  Grey Level Variance (GLV)  Dependence Variance (DV)  Dependence Entropy (DV)  Low Grey Level Emphasis (LGLE)  High Grey Level Emphasis (HGLE)  Small Dependence Low Grey Level Emphasis (SDLGLE)  Small Dependence High Grey Level Emphasis (SDHGLE)  Large Dependence Low Grey Level Emphasis (LDLGLE)  Large Dependence High Grey Level Emphasis (LDHGLE) |

**Table S8**. Table of PyRadiomics features used in analysis.
